# Expenses of Administration

**Published:** 1913-03-15

**Authors:** 


### OUR NATIONAL INSURANCE ACT DEPARTMENT.

### XLVI.—Expenses of Administration.

Many problems concerning the National Insurance Act which have been agitating the minds of those who are in daily contact with it in its practical application are now beginning to demand solution, and will before long command public attention.

One of the most important of these problems is the regulation by the Approved Societies of their administrative expenses to come within the maximum allowance prescribed by the Insurance Commissioners.

Another cognate problem which is bound to attract considerable attention in the near future is in regard to the total cost to the nation of this latest experiment in social legislation.

We propose this week to examine these questions in turn.

### The Expenses of Approved Societies.

Dealing first with the administrative expenses of the Approved Societies, we would point out that there is no provision in the Act itself as to the amount to be allowed to the Approved Societies for management expenses, but they are required to keep administration accounts quite separate and distinct from the contribution and benefit accounts, and the expenses must be strictly regulated to the amount prescribed by the Insurance Commissioners. Furthermore, the accounts are subject to audit by Government auditors, who only have power to sanction expenditure properly incurred, and who must see that no part of the money applicable to benefits is spent on expenses of management.

According to the actuarial estimates the sum allowed for expenses in normal cases is 3s. 8d. per member per annum, and this is the sum which was originally prescribed by the Insurance Commissioners as the maximum limit of expenditure in a single year. This sum had to cover not only all the expenses directly incurred by the Societies in the form of remuneration to officers, office rent, printing, stationery, and postages, but had also to meet the costs of administration incurred on behalf of the members by the Insurance Committees on account of medical and sanatorium benefits. As the Societies had no means of telling what the expenses of the Insurance Committees would be, the regulations have been altered so that an average sum of 3d. per member per annum is to meet these expenses. This leaves a sum of 3s. 5d. per member for the direct expenses of the Approved Society. In the first quarter an additional sum of 1s. was allowed for preliminary expenses, but there is no intention, of course, to repeat this allowance. In regard to this special preliminary grant, we understand that some of the smaller Approved Societies, which were desirous of increasing their membership, actually paid away half of this sum as an introductory fee; thus the extraordinary expenditure of the first quarter must have had to be cut down in some other way. The amount of 3s. 8d. per annum is equivalent, after making allowance for arrears, to 13 per cent. of the contributions in the case of men and 15 per cent. in the case of women. These allowances appear at first sight to be fully adequate, but those who have intimate knowledge of the amount of work required in carrying out the complex provisions of the Act are already expressing doubt on the point. It should be pointed out that this sum of 3s. 8d. includes the State proportion of two-ninths in the case of men and one-quarter in the case of women.

If the Approved Societies are unable to regulate their expenditure within the prescribed limits, they must immediately take steps to rectify the matter, either by making payments from other funds or by making a levy on the members for the purpose. It is laid down in the regulations that each quarter must be, as it were, a watertight compartment in regard to the expenses of that quarter. That is to say, if any saving is made on account of expenses of management in one quarter, that saving is not allowed to be credited to the next quarter, should the expenses of that quarter exceed the allowance. Any savings on account of expenses will go towards increase of benefits, so that a Society which is consistently able to carry out its operations at a lower figure than the amount prescribed will be at an advantage as compared with those Societies which spend to the full limit. It was hoped that in the larger Societies the very magnitude of their operations would enable them to curtail the expenses to a minimum, and this, no doubt, will be the case as compared with the smaller Societies. At the same time, we already hear that the representatives of a number of the largest Societies are dissatisfied with the remuneration they are receiving for the work they are required to carry out. Having knowledge of their duties and the amount which they receive in payment therefor, the contention seems to us to be a reasonable one, but there is no prospect of being able to increase their remuneration whilst the limits of expenditure allowed by the Commissioners remain unaltered. These representatives of the larger Approved Societies have been content so far, because they have realised the urgency of the work, and the necessity of carrying it through despite all personal inconvenience. The time has, however, now come when they are beginning to see that the work demanded of them is more complex than they had any idea of at the outset, and there is no doubt that they will before long demand a higher recognition of their services. Thus it is clear that the question of administrative expenditure on behalf of the Approved Societies is one which will call for very careful consideration in the near future.

Total Cost to the Nation.

Turning now to the other problem indicated at the commencement of this article—namely, the total cost to the nation of the National Insurance Act—we have still to wait until the introduction of the Budget before we are able to state authoritatively what the charges will be. The introduction of the Budget is this year promised at an early date, and it is being freely stated that the Estimates will reach the colossal and unprecedented total of £200,000,000, involving a large increase in revenue on account of the National Insurance Act and its forerunner, the National Old Age Pensions Act. Until the accounts for the year are presented, it is impossible to say what amounts have already been spent and what larger charges there may be still in store for the future, but it is patent to all who have an intimate knowledge of things as they are, that the total cost will very considerably exceed the original estimate. This does not mean that the financial basis of the system is unsound, or that the actuarial calculations are incorrect upon which the relations between benefits and contributions were founded. The financial basis is demonstrably sound, and is supported by the authority of the best actuarial talent that the Government could obtain, whilst the estimates of the numbers who would become insured under the provisions of the Act have been very closely realised. It is not, therefore, on this ground that there is reason to expect a departure from the estimates, but rather on account of the much greater expenditure on purely administrative work than had been anticipated. In any new enterprise there is of necessity an outlay of capital in the nature of establishment expenses. These expenses do not recur, and are incidental only to the initial stages of the undertaking. Expenditure of this character had, of course, to be incurred in the setting up of a system of National Insurance, and no valid objection can be raised to a reasonable expenditure of this kind. Due allowance for this fact will have to be made when the first accounts are submitted, but, when the most ample allowance has been taken into consideration for establishment expenses, it is to be feared that the annual administrative charges will be vastly greater than the nation were led to expect. We do not say that the salaries of the Insurance officials and of their clerks are too high for the work they have to do; on the contrary, judging at least by the amount of work they have had to do during the past twelve months, and their long office hours, we are of opinion that their remuneration has been none too adequate. What we do say, however, is, that closer attention should have been given to the question of costs of administration before the Act was put into operation. The experiment—for it is nothing more—might have been tried in the first place on a smaller scale, so that experience could have been obtained before the final and complete scheme was adopted. Criticism after the event is easy, and it is unnecessary to elaborate on the reasons for the hasty legislation and the equally hasty commencement of its operation, for it is well known that there were urgent political causes which rendered this haste imperative. What is more to the point, is to express the opinion, that the sooner the system is simplified in its details and in the amount of official machinery involved in carrying it out, the better it will be for all concerned. One of the most fruitful suggestions that can be made in this connection is in regard to the present system of four Commissions. The institution of these four separate bodies has required the setting up of a fifth Joint Committee to co-ordinate their work, and it certainly seems that very much time and overlapping of work could be saved by the concentration of the Commissions in a single authority.

